# Dynamic Changes in Brain Functional Connectivity during Concurrent Dual-Task Performance

**DOI:** 10.1371/journal.pone.0028301

**Published:** 2011-11-29

**Authors:** Luca Cocchi, Andrew Zalesky, Ulrike Toepel, Thomas J. Whitford, Marzia De-Lucia, Micah M. Murray, Olivia Carter

**Affiliations:** 1 Melbourne Neuropsychiatry Centre, The University of Melbourne, Melbourne, Australia; 2 Queensland Brain Institute, The University of Queensland, Brisbane, Australia; 3 Radiology Department, Centre Hospitalier Universitaire Vaudois and University of Lausanne, Lausanne, Switzerland; 4 Neuropsychology and Neurorehabilitation Service of the Department of Clinical Neuroscience, Centre Hospitalier Universitaire Vaudois and University of Lausanne, Lausanne, Switzerland; 5 EEG Core, Center for Biomedical Imaging of Lausanne and Geneva, Lausanne, Switzerland; 6 Psychological Sciences, The University of Melbourne, Melbourne, Australia; University of Granada, Spain

## Abstract

This study investigated the spatial, spectral, temporal and functional proprieties of functional brain connections involved in the concurrent execution of unrelated visual perception and working memory tasks. Electroencephalography data was analysed using a novel data-driven approach assessing source coherence at the whole-brain level. Three connections in the beta-band (18–24 Hz) and one in the gamma-band (30–40 Hz) were modulated by dual-task performance. Beta-coherence increased within two dorsofrontal-occipital connections in dual-task conditions compared to the single-task condition, with the highest coherence seen during low working memory load trials. In contrast, beta-coherence in a prefrontal-occipital functional connection and gamma-coherence in an inferior frontal-occipitoparietal connection was not affected by the addition of the second task and only showed elevated coherence under high working memory load. Analysis of coherence as a function of time suggested that the dorsofrontal-occipital beta-connections were relevant to working memory maintenance, while the prefrontal-occipital beta-connection and the inferior frontal-occipitoparietal gamma-connection were involved in top-down control of concurrent visual processing. The fact that increased coherence in the gamma-connection, from low to high working memory load, was negatively correlated with faster reaction time on the perception task supports this interpretation. Together, these results demonstrate that dual-task demands trigger non-linear changes in functional interactions between frontal-executive and occipitoparietal-perceptual cortices.

## Introduction

Different brain regions are now recognized for their remarkable functional specificity. However, our survival and success in everyday life depends on our ability to rapidly coordinate and integrate these distinct operations [1], [2]. While it has been demonstrated that the addition of multiple task demands can lead to impaired performance, the extent to which the brain is able to cope with the concurrent processing demands of multiple independent tasks appears to depend on the nature of the tasks. Indeed, in some instances a clear cost of dual-task is apparent (for a review see [3]) whereas in other cases the specific changes in the brain dynamics associated with dual-task performance does not affect, or even improves, the execution of the second task [4], [5], [6]. One general conclusion that can be drawn from the disparate set of results reported to date, is that the functional consequences of a first task on second task performance are difficult to summarize in terms of a simple linear relationship between executive and sensory-perceptual processes, and are likely to depend on complex brain dynamics.

In a previous study, we analysed electroencephalography (EEG) data acquired during an experiment in which subjects were asked to perform a visual perceptual task while concurrently maintaining an unrelated set of information in visual-spatial working memory (VSWM) [5]. In line with recent functional magnetic resonance imaging (fMRI) studies, our previous analysis of phase-locked brain activity (event-related potentials) revealed that occipital, parietal and frontal cortices play a central role in mediating the functional interplay between sensory and executive operations during dual-task performance [7], [8], [9]. The rationale behind the present study was to use a novel data-driven method to identify changes in functional relationships (functional connectivity) between brain regions during either single- to dual- task transition or when dual-task demands are increased. Specifically, we sought to characterize the spatial and temporal proprieties of changes in statistical dependencies (coherence) among remote neurophysiological events in distinct frequency bands. We also aimed to provide behavioural evidence for the functional relevance of hypothesized changes in brain dynamics related to dual-task performance. Notably, the location of these changes in functional relationships was not biased by a priori assumptions. Our new analysis is based on a recent approach to assess functional connectivity using fMRI data [10]. A similar analysis strategy was also recently applied successfully to EEG data to characterise functional interactions between distinct regions within large-scale cortical networks [11]. Compared to previous approaches the one adopted here allowed the analysis of changes in brain functional connectivity in isolated pairwise connections, providing the best chance to describe the brain dynamics supporting dual-task performance and complement results from our previous investigation [5].

## Materials and Methods

### Participants

Eleven (3 female) healthy right-handed individuals [25–41 years; mean ± standard deviation (SD) = 30.4±4.8 years] participated in the study (see Experiment 2 in [5]). All subjects were participating voluntarily and gave their informed written consent before starting the experiment. The procedures were approved by the Ethics Committee of the Faculty of Biology and Medicine (University of Lausanne, Switzerland) and the study was conducted in accordance with the Declaration of Helsinki (http://www.wma.net/en/30publications/10policies/b3/index.html).

### Experimental paradigm and procedure

**Figure 1:**
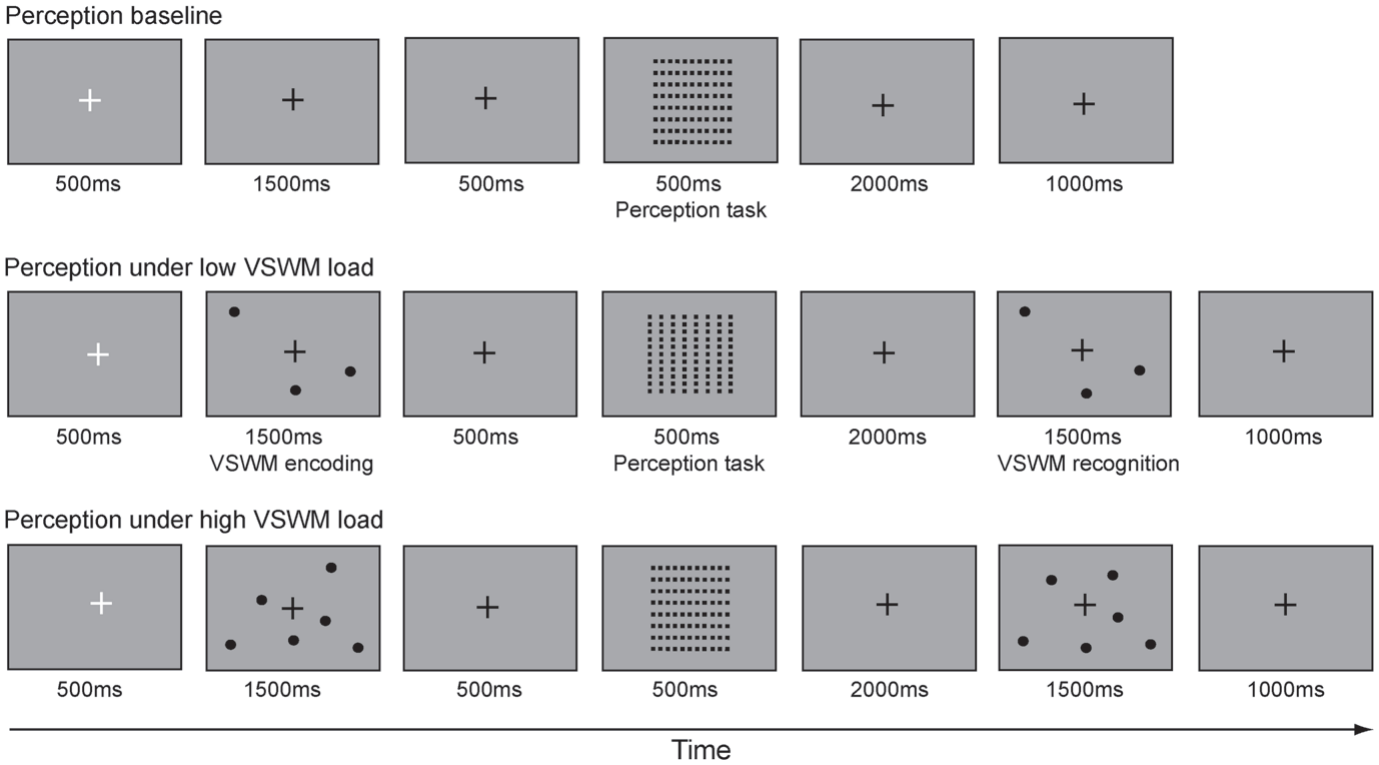
Experimental design. Visual-spatial working memory (VSWM) stimuli consisted of black disks (6 for imposing high loads and 3 for low loads) presented on a grey background. Subjects were asked to encode (1500 ms) the location of each disk and. After a delay of 3000 ms, a new set of disks were presented and subjects were asked to report whether or not all disks were in the same location. The perceptual stimuli (second task) consist in a grid composed by black squares. Subjects had to indicate if the squares were organized in column (see the low VSWM load condition) or row (see the baseline and high VSWM condition). Perceptual stimuli were presented in 50% of the dual-tasks conditions.

A full description of the paradigm may be found in Cocchi et al. [5]. Briefly, participants were required to concurrently perform a VSWM and a basic visual perceptual task (see Figure 1). Specifically, participants were told to perform two separated unrelated tasks. One task was to remember an array of black disks. It was emphasized to participants that it was important to remember the specific location of each disk. Indeed, in a following trial phase they would be presented with a second array of disks and their task was to determine if any of the original disks' locations had moved. For the second task participants were presented with a grid of black squares and they were asked to discriminate whether it was arranged in rows or columns. It was explained to participants that throughout the experiment they would be presented with (i) the disk memory task alone; (b) the grid perception task alone or (iii) both tasks, with the grid task presented during the memory task's delay period (between the first and second presentation of the disks arrays). A standard practice session was administered to subjects to ensure that they understood the paradigm and the procedure. The beginning of each new trial was indicated by the appearance of a white fixation cross. Participants were asked to perform both VSWM and visual perceptual tasks as fast and as accurately as possible.

Task stimuli were presented on a 21 inch monitor and viewed from a distance of 80 cm. The VSWM task was comprised of stimulus encoding, retention and recognition phases. In the encoding phase either 0, 3, or 6 black disks (indicating no, low and high VSWM load) were presented against a grey background. Disks were always presented in random and non-overlapping positions occupying up to 90% of the screen dimension to guarantee a similar attention focus across conditions. During the encoding phase, the disks were presented for 1500 ms and then removed. After a 3000 ms retention period, the disks were presented again during a 1500 ms recognition phase. For 50% of the recognition trials, 3 disks were displaced (∼2.0° visual angle) in a random direction from their original location. Subjects were asked to indicate, via key-press, whether the disks were in the same or different location in the second presentation.

The visual-perceptual task was adapted from Kurylo et al. [12]. Subjects were presented with a grid of 432 black squares on a gray background (∼12° visual angle), in 50% of the VSWM task trials. The grid was presented for 500 ms during the VSWM retention phase; 500 ms after the presentation of the first disk array in the VSWM task. To create the perceptual effect of row or columnar organization based on the principle of visual proximity [13], the horizontal and vertical distance between arrangements of the squares was uniformly manipulated (∼0.15° visual angle). The arrangement of the grid into either columns or rows was randomly assigned with equal probability across trials. Subjects were required to report, via key-press, whether the grid was organized in rows or columns (see Figure 1).

To test the effect of changes in VSWM load on the concurrent grid perception, we investigated three trial conditions: (i) perceptual task alone; (ii) perceptual task under low VSWM load and (iii) perceptual task under high VSWM load. Overall, participants completed 120 trials per condition during a single experimental session that lasted approximately 90 minutes (12 blocks of 10 trials for each condition).

### EEG signal acquisition

Continuous EEG was acquired in an electrically shielded sound-attenuated cabin at 1024 Hertz (Hz) with a 160-channel Biosemi ActiveTwo system (Biosemi, Amsterdam, Netherlands). During data acquisition, all electrodes were connected to the system's internal loop- i.e., Common Mode Sense (CMS) and Driven Right Leg (DRL) electrodes, which functions as a feedback loop driving the average potential across the electrode montage to the amplifier zero (for details see http://www.biosemi.com/faq/cms&drl.htm). The EEG was online band-pass filtered between 0.1 and 100 Hz.

### Data Analysis

#### EEG analyses

Analyses were performed using Cartool software (http://sites.google.com/site/fbmlab/cartool) and additional customized MATLAB scripts. Connectivity analyses were performed on the Neuropsychiatry Imaging Compute Cluster (NICC), consisting of 9 Dell PowerEdge R410 (Dual Intel Quad Core 2.26GHz E5520 Xenon CPU, 16GB RAM) work nodes. Each node is a Dell PowerEdge R410 (Dual Intel Quad Core E5520 Xenon, CPU 2.26GHz, 8Mb Cache, 5.86 GT/s QPI), for a total of 8 cores per node. Epochs of subjects EEG recordings over the -100 to 500 ms period relative to the onset of the visual grid were averaged for each correctly answered trial (∼110 trials for each condition). EEG epochs with ocular artifacts were semi-automatically identified and rejected on the basis of the horizontal and vertical electrooculogram recordings, and a rejection criterion of ±80 µV for all electrodes. Data from artifact electrodes were interpolated using 3 dimensional splines [14]. Data were band-pass filtered from 0.1–40 Hz. Subjects' averaged visual evoked potentials (VEPs) were calculated as a function of whether the grid was presented within a no load, low VSWM load and high VSWM load context. Data were recalculated to the common average reference prior to group-averaging, and baseline correction was applied to the 100 ms epoch preceding the grid stimulus.

We estimated the sources in the brain underlying VEPs for each subject and condition using a distributed linear inverse solution (ELECTRA), calculated on the basis of the local autoregressive average (LAURA) regularization approach [15]. The current implementation of LAURA was generated with the Spherical Model with Anatomical Constraints (SMAC, [16]). The inverse solution space was down-sampled (adopting algorithms similar to that described in [17]) from the original resolution of 3005 nodes to 280 nodes, equally distributed within the gray matter of the Montreal Neurological Institute's average brain. The reduction of the analysis space to 280 nodes was necessary due to computational limits. Several studies have documented and discussed in detail the spatial accuracy of this inverse solution [15], [18], [19], [20], [21].

The extent of coherence between every possible pair of nodes [(280 × 279)/2)] was estimated using the Morlet wavelet (for mathematical details see section 3.4 in Grinsted et al. [22]; http://www.pol.ac.uk/home/research/waveletcoherence/). Coherence yields a time- and frequency- resolved estimate of the phase and amplitude consistency between the respective time courses of a pair of nodes. Coherence was sampled in the time domain at the native EEG sampling rate over the entire duration of the 500 ms visual perception task, and in the frequency domain across 28 logarithmically scaled frequencies spanning approximately 8 to 40 Hz. A time-averaged coherence estimate [23] was then calculated for each of three non-overlapping frequency bands: alpha (10–13 Hz), beta (18–24 Hz) and gamma (30–40 Hz). This calculation was repeated independently for each of the three distinct VSWM load conditions (no, low and high VSWM), thereby yielding a separate time-averaged coherence estimate for each of three frequency intervals and each of three load conditions. Frequency bands were chosen a priori on the basis of recent studies showing that visual perceptual [11] and visual working memory [24] processes involve changes in coherence/phase synchrony in fronto-parietal-occipital connections in the beta frequency (∼18–24 Hz). In addition, changes in phase synchrony in the alpha (10–13 Hz) and gamma (30–40 Hz) frequencies have also been associated with visual working memory maintenance and load changes [24]. The choice of a priori frequency bands was also dictated by the high computational demands of the analyses. Pairs of nodes separated in distance by less than 40 mm were excluded a priori to alleviate spurious coherence arising from volume conduction effects, leaving a total of 10,861 node pairs for consideration (between the 280 nodes) [25], [26]. The 40 mm constraint was implemented by computing the Euclidean distance between every possible pair of nodes and then omitting any nodes for which the Euclidean distance was less than 40 mm. The distribution of coherence estimates across subjects was found to be normal, thereby justifying the use of subsequent parametric statistical testing (Kolmogorov–Smirnov tests, p>0.05). With regard to impact of volume conduction on the quantification of coherence, we have taken a strategy based in part on the recommendation of Schoffelen and Gross [25] to focus on experimental contrasts where volume conduction effects should apply similarly to all conditions. Additionally, these authors also recommend quantifying coherence on the source rather than sensor level as an additional means of avoiding confounds due to volume conduction while also providing more detailed inference about localization (see also similar approaches in dynamic causal modeling applied to EEG/MEG; e.g., [27]).

A within-subjects one-way ANOVA was calculated for each of the distinct pairs of nodes to test for a change in the extent of time-averaged (0–500 ms) coherence between the three VSWM load conditions (i.e., perception under no load, low and high load). Each pair of nodes was therefore endowed with an *F*-statistic and corresponding *p*-value. Considering every possible pair of nodes ensured a completely data-driven approach that was free of any regional bias.

A clustering method, called spatial pairwise clustering (SPC), was then used to pinpoint clusters of nodes between which the extent of coherence was significantly changed as a function of VSWM load. SPC is a close descendant of the network-based statistic (NBS) [10] and differs only in the use of a *pairwise* clustering criterion. The NBS and SPC are complimentary procedures, each offering unique advantages and disadvantages. In general, SPC is suited to the detection of isolated connections, whereas the NBS is suited to the detection of interconnected networks. An advantage of SPC is that the null hypothesis can be rejected separately at the resolution of individual connections. In contrast, with the NBS, the null hypothesis can only be rejected at the resolution of an entire network, but not for the individual connections comprising that network. This improved localizing resolution of SPC was the primary motivation for its use in the present study.

SPC was implemented as follows: Any pair of nodes with an *F*-statistic exceeding a threshold of 8 was admitted to a set of supra-threshold node pairs. The F-statistic threshold of 8 was determined using a process of trial-and-error, which is generally the case in such analyses, given that definitive criteria for selecting a threshold are not available. Note that control of Type I error is guaranteed irrespective of the choice of threshold. A search was then conducted to identify clusters of *pairwise* neighbors contained in the set of supra-threshold node pairs. Two nodes *x_1_* and *x_2_* were considered neighbors if the distance by which they were separated was less than 7 mm. Furthermore, if *x_1_* was a neighbor of *x_2_* and *y_1_* was a neighbor of *y_2_*, node pairs (*x_1_,y_1_*) and (*x_2_,y_2_*) were said to be pairwise neighbors and shared a pairwise relation. Several distinct clusters of pairwise neighbors were identified in the set of supra-threshold node pairs.

The size of a pairwise cluster was defined as the number of pairwise relations it comprised. Permutation testing was used to attribute a *p*-value to each pairwise cluster based on its size. Five thousand permutations were generated in which the labels of the three load conditions were randomly exchanged for each node pair. The size of the *largest* pairwise cluster was recorded for each permutation, resulting in an empirical null distribution for the largest pairwise cluster size. Recording the size of the largest pairwise cluster in the permuted data ensured weak control of the familywise (FWE) error rate [28], [29]. Finally, a corrected *p*-value for a pairwise cluster of size *k* identified in the un-permuted data was calculated as the proportion of permutations for which the largest pairwise cluster was greater than or equal to *k*.

Note that the dependencies induced by volume conduction effects are likely to be preserved in the permuted data, assuming that the extent of volume conduction is homogenous across the three load conditions. Therefore, the pairing of two neighboring nodes separated by less than 7 mm is unlikely to be attributable to volume conduction alone, since permutation testing does not eradicate dependencies induced by volume conduction. More specifically, if two neighboring nodes separated by less than 7 mm are clustered in the observed data as a consequence of dependencies induced by volume conduction, they are also likely to remain clustered in the permuted data. In contrast, if they are clustered as a consequence of differences in coherence associated with changing VSWM load, they are unlikely to remain clustered in the permuted data. Permutation testing involves shuffling the three load conditions, which exclusively tests for differences in coherence associated with changing VSWM load.

This entire procedure was repeated independently for each of the three frequency bands considered.

Four pairwise clusters satisfying statistical significance (p<0.05, FWE corrected) were identified, each of which will be referred to as a *functional connection* in this paper from now on. All functional connections comprised two spatially distinct clusters of nodes. The cluster-averaged time series were used to display and analyse change in coherence as function of VSWM load during grid perception in each cluster of interest. Finally, the evolution of coherence within each connection was plotted as a function of time and the significance of change in coherence was then assessed in specific time subintervals of interest using within-subjects ANOVA. This analysis utilized a time-resolved estimate of coherence (500 estimates) derived from the cluster-averaged time course, whereas the main analysis utilized time-averaged coherence.

Coherence can be driven by either consistent phase differences between two signals or correlation in their amplitudes. While it is difficult to disambiguate phase synchrony from amplitude effects, no significant change in average signal power across the three VSWM load conditions was noted in any of the four connections identified (Figure S1). This suggests amplitude effects were not the predominant driver of the observed changes in coherence. The integral of the power spectral density (PSD) over the relevant frequency bands was computed to estimate average signal power. The periodogram with a Hamming window was used to estimate the PSD. To disambiguate amplitude effects, an alternative would have been to normalize with respect to amplitude in the frequency domain [30]; however, the benefit of this normalization has been questioned [31].

## Results

### Behavioral results

#### Perceptual task

As reported previously [5], a within-subjects one-way ANOVA on reaction times (RTs) indicated a significant main effect of VSWM load (F_(2,20)_ = 7.14, p<0.01). Participants were faster in the context of a high VSWM load (mean: 871 ms) as opposed to either a low VSWM load (890 ± contrast's s.e.m. 5 ms; t_(10)_ = 2.32, p = 0.04) or baseline (908±9 ms; t_(10)_ = 3.55, p<0.01). Accuracy (∼90% in each condition) and sensitivity (d-prime, [32]) were high and did not differ significantly between conditions (accuracy: F_(2,20)_ = 1.00, p = 0.38; sensitivity: F_(2,20)_ = 1.03, p = 0.37).

#### Visual-spatial working memory task

Within-subjects two-way ANOVA on RTs showed a significant main effect of VSWM load (F_(1,10)_ = 20.58, p = 0.01) and condition (F_(1,10)_ = 10.59, p<0.01), as well as an interaction between load and condition (F_(1,10)_ = 9.58, p = 0.01). Subjects showed slower RTs in high VSWM load trials compared to low load trials (low load: average 896 ms; high load: 944 ms). In addition, dual-task trials were associated with increased RTs compared to single-task trials (single-task: average 895 ms, dual-task: 945 ms). In term of task accuracy only a significant main effect of condition was found (F_(1,10)_ = 53.25, p<0.01), where decreased accuracy was associated with the dual-task trials (single-task: average 78%, dual-task: 69%).

### Brain dynamics

#### Functional connections showing global changes in coherence during grid perception as a function of VSWM load

**Figure 2:**
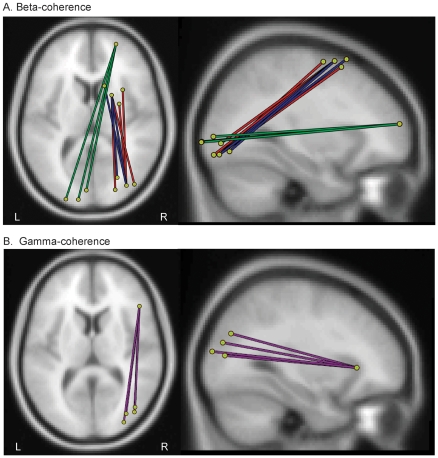
Functional connections showing changes in coherence as a function of concurrent working memory load during visual perception. Spatial pairwise clustering (SPC) was used to identify functional connections in which coherence was modulated as a function of concurrent working memory load during a grid perception task. Beta-connections (18–24 Hz) are displayed in red, blue and green (panel A) and the gamma-connection (30–40 Hz) displayed in purple (panel B). All connections satisfy p<0.05, familywise error (FWE) corrected.

Average changes in beta-coherence were found in three connections during the perceptual task as a function of VSWM load (Figure 2A and Table S1). Two connections [connections 1 (red) and 2 (blue) in Figure 2A] showed a similar spatial structure encompassing dorsofrontal and occipital cortices in the right hemisphere. A third connection encompassed the right prefrontal (Brodmann's area 10) and left occipital cortices (green connection in Figure 2A). Gamma-coherence changed across conditions in a single connection encompassing the inferior frontal gyrus/frontal operculum and occipital/inferior parietal cortices within the right hemisphere (connection 4; Figure 2B and Table S1). No significant differences in coherence were observed for frequencies in the alpha range (10–13 Hz). In addition, average coherence in each frequency band of interest was similar over the baseline period preceding the perceptual stimulus (grid) onset (i.e., -100 ms to 0).

**Figure 3:**
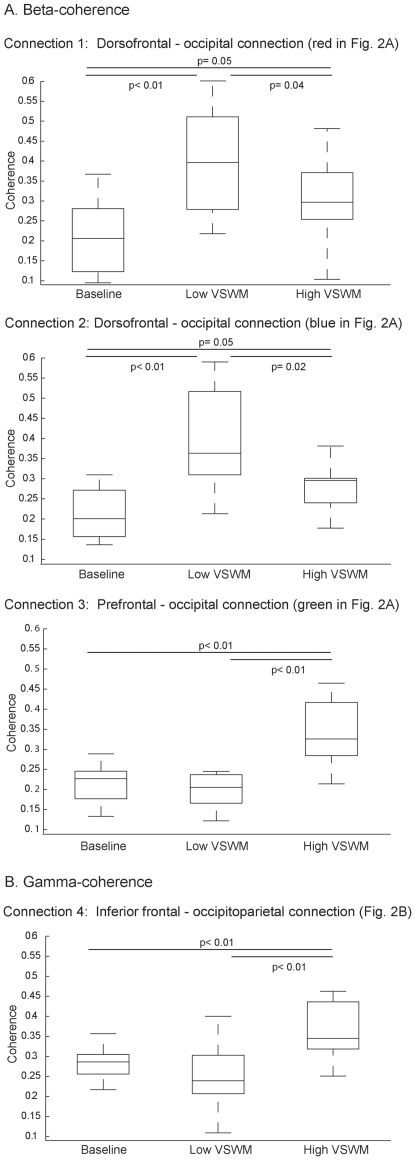
Changes in coherence as a function of working memory load during grid perception. Pairwise comparisons (*t*-tests) were computed to assess time-average (500 ms of grid perception) fluctuations in coherence across conditions: baseline, low and high visual-spatial working memory (VSWM) load. Panel A: beta- connections (18–24 Hz); panel B: gamma-connections (30–40 Hz). Box Plots depicts 50^th^, 75^th^ and 25^th^ percentiles of coherence in each condition. Top and bottom lines extending from the box represents the highest and lowest values of coherence that are not outliers (>1.5 the interquartile range).

In the dorsofrontal-occipital beta-connections (connections 1 and 2; Figure 2A), coherence during grid perception was highest in the low VSWM load trials. However, both high and low load trials showed greater coherence relative to baseline (*p*≤0.05 for all post-hoc comparisons; Figure 3A). In contrast, in the third prefrontal-occipital beta connection, coherence was only elevated in the high VSWM load trials, while there was no significant difference between baseline and low VSWM load trials (Figure 3A). The same changes in average coherence as a function of VSWM load were observed in the gamma-connection (connection 4; Figure 3B).

#### Evolution of coherence as function of grid perception time

**Figure 4:**
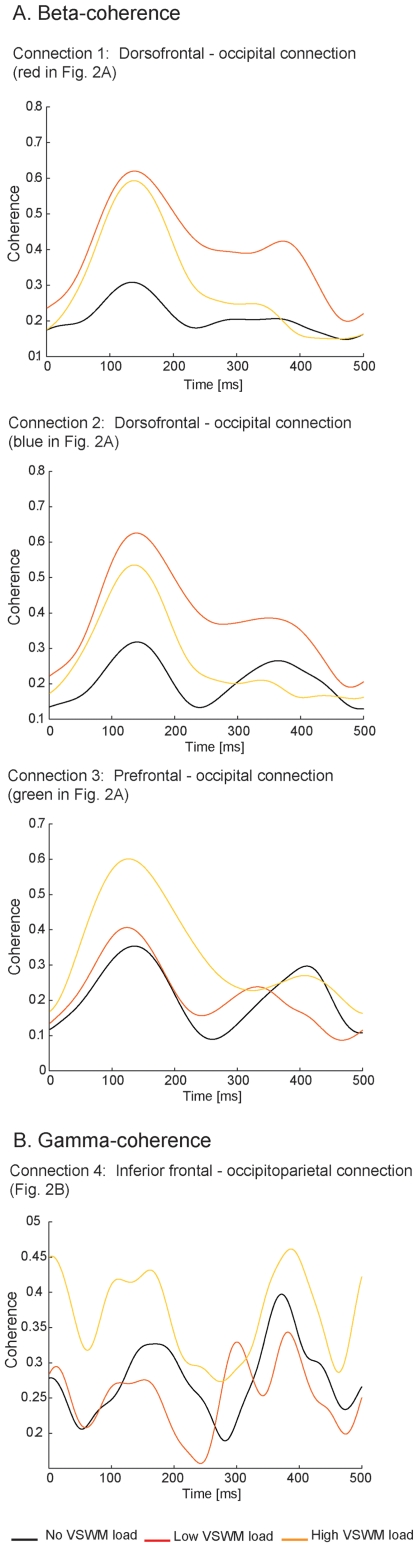
Evolution of coherence as a function of time during grid perception. Change in coherence in the four functional brain connections of interest as a function of time during grid perception. Black lines: baseline trials; orange lines: low VSWM load trials; yellow lines: high VSWM load trials.

Coherence evolved similarly for beta-connections 1 and 2 during the 500 ms of grid perception (Figure 4A). Between 100–200 ms post grid onset coherence levels differed across conditions (p<0.01; Figure S2). However, the fact that average coherence was highest in low VSWM load trials compared to both baseline and high load trials appears to be mainly due to sustained coherence in a later period of grid perception (250–400 ms post stimulus onset, p<0.01; Figure 4A and Figure S2). In the third beta-connection, the general increase in coherence in high VSWM trials, compared to low and baseline trials, was mostly due to coherence fluctuations in the relatively early phases of stimuli processing (first 200 ms post grid onset, p<0.01; Figure 4A and Figure S2). Changes in coherence during grid perception in the gamma-connection (connection 4; Figure 4B) showed a more complex behaviour, with a broad increase in the connection's coherence in high VSWM load trials compared to both baseline and low load trials across the 500 ms analysis period.

#### Relation between changes in coherence and behaviour

**Figure 5:**
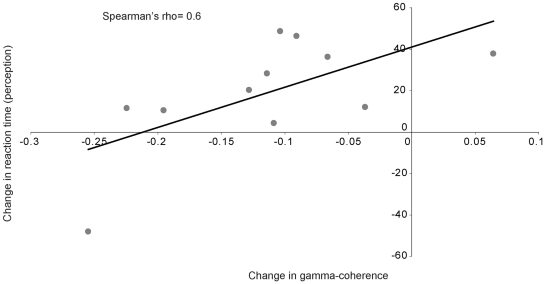
Gamma-connection: Relation between changes in coherence and reaction time in the visual-perceptual task as a function of working memory load (low vs. high). Correlation between changes in time-average (perceptual stimulus presentation period) gamma-coherence from low to high working memory load, and relative changes in grid task reaction times (ms).

Finally, we considered whether the change in performance on the perceptual or VSWM tasks were associated with differences in time-averaged coherence between low and high VSWM trials during the 500 ms grid presentation. The magnitude of coherence change in the gamma-connection was positively correlated with the relative reduction of RT in the visual perceptual task as a function of VSWM load (Spearman's rho = 0.6, p = 0.03; Figure 5). No significant correlations were found between behaviour and brain coherence in the other three connections.

## Discussion

In this study we applied a data-driven procedure to characterize the spatial, spectral and temporal properties of functional connections between distal brain regions involved in the concurrent execution of unrelated visual perception and working memory tasks. Significant changes in beta and gamma coherence associated with concurrent VSWM and perceptual demands were identified in four cortico-cortical connections. These functional connections encompassed frontal, parietal and occipital cortices and showed distinct coherence profiles across condition trials and time. Together, these results suggest that the demand of our dual-task paradigm triggered a non-linear reorganization of the functional interactions between frontal-executive and occipitoparietal-perceptual brain regions. These findings are also in line with our previous analysis showing that high VSWM load enhance frontal brain activity while concurrently down regulating occipital and parietal activity [5]. However, the current findings extend our previous results by suggesting that the observed co-activation pattern is related to non-linear changes in functionally distinct cortico-cortical connections.

Three distinct functional brain connections were identified within the beta frequency band (18–24 Hz) and one in the gamma frequency band (30–40 Hz). While other connections are likely involved in VSWM and perceptual processing, the four connections isolated here are unique in that they were the only connections to show a significant modulation in coherence with either the addition of a second working memory task or the increase in load within the dual-task context. Together the four functional brain connections encompassed dorsal and ventral frontal, inferior-parietal and occipital cortices, mainly in the right hemisphere. These results are consistent with previous fMRI and EEG findings implicating these regions in top-down cognitive control [33], [34], [35], visual attention [36], [37], working memory [24], [38] and dual-task performance [5], [8], [9]. Our results are also in line with recent observations suggesting that large-scale functional interactions between frontal, parietal and occipital cortices in the beta and gamma frequency bands are critical for visual perception, attention and working memory functions [11], [24], [39]. While previous studies provide valuable information about specific brain regions involved in dual-task processes [7], [8], [9], ours is the first study to directly assess how functional connectivity (coherence) between different brain regions and over specific latencies change during a single-task compared to a dual-task situation and/or when task demands are increased within the same dual-task context.

The addition of a VSWM task resulted in two distinct patterns of change within the four functional connections. In beta-connections 1 and 2, which encompassed dorsofrontal and occipital regions, coherence increased from the single-task to dual-task condition. In contrast, beta-connection 3 (encompassing prefrontal and occipital cortices) and gamma-connection 4 (encompassing inferior frontal gyrus/frontal operculum and occipitoparietal regions) were not affected by the addition of the second task. Considering the effect of increasing VSWM load within the dual-task context, a different pattern of functional reorganization also became apparent. Specifically, coherence within beta-connections 1 and 2 decreased as a function of VSWM load. The opposite result was found within beta-connection 3 and gamma-connection 4, which showed a significant increase in coherence from the low to high load. Together these results suggest that these connection pairs may have distinct functional roles in managing the dual-task demands imposed by our paradigm.

To further explore the nature of functional connectivity changes within the four cortico-cortical connections of interest we considered time-resolved coherence fluctuations across the 500 ms window of dual-task performance. In connections 1 and 2, beta-coherence was not sustained at high levels throughout the dual-task period, but rather decayed considerably approximately 250 ms after presentation of the visual perceptual stimulus (grid). As these connections encompass dorsofrontal and occipital regions, which are known to be central to visual working memory maintenance [40], [41], the observed reduction in coherence in the later portion of the time window may reflect a functional breakdown of the connection. This hypothesis is consistent with the observed impairment in VSWM performance under high VSWM load.

In terms of beta-connection 3, coherence between occipital and prefrontal regions during performance of the perceptual task was unaffected by the dual-task requirements of the low load. It was only under the high load that coherence between these distinct brain regions increased. This result is in line with previous data suggesting a key role of rostrolateral prefrontal cortex in multitasking [42], [43] and with an influential theoretical model proposing that this part of human cortex is specifically involved in holding different goals online while implementing secondary goals [35], [44], [45]. As changes in coherence appeared to be limited to the initial 200 ms following grid presentation, the enhanced coherence may support the facilitated processing of concurrent task-relevant perceptual stimuli by conveying early top-down modulatory signals from rostral prefrontal brain regions [33], [35], [46], [47]. While this hypothesis remains to be tested, our results suggest that when executive resources are saturated prefrontal top-down signals may facilitate the processing of new relevant information. A similar pattern of results was found in the gamma-connection, with increased coherence only seen after the addition of the high VSWM load. Interestingly, the change in gamma coherence from low to high VSWM load was negatively correlated with the observed increased speed of RT on the visual perception task. Specifically, individuals that showed the greatest difference in coherence between low and high load trials showed the smallest effect of increased VSWM load on the RT for the perception task. This pattern suggests that the gamma-connection may protect early visual processing from the influence of increased VSWM load. This finding supports current speculation that the inferior lateral prefrontal cortex play a role in interference control and more generally in coordinating top-down control in the context of managing concurrent independent task objectives ([48], for an overview see [46]). Specifically, this result emphasize that the observed perceptual facilitation as a function of VSWM load is related to a series of complex changes in functional connectivity in distinct cortical connections.

This study has some limitations that need to be considered in interpreting the results. First, while precautions were taken to reduce the effect of volume conduction on the results, this potential confound cannot be wholly excluded. Functional connectivity analyses (SPC) were performed using average data time-locked to stimulus onset. This procedure has most likely resulted in a loss of sensitivity to detect changes in coherence. However, coherence changes as a function of VSWM load were detected in both beta and gamma frequency bands. These positive results indicate that we had sufficient signal power to statistically detect changes in coherence, whilst maintaining strong control of type I error. Also, no coherence differences were detected in the baseline period preceding the perceptual stimulus onset (in any frequency band). Thus, we contend that the observed changes in beta and gamma coherence were evoked by the perceptual (grid) stimulus. Further, current connections showing significant coherence modulation as a function of VSWM load overlap with the global network identified by our previous electrical neuroimaging approach combining topographic clustering with source estimations [5]. While based on different theoretical assumptions, the results of this previous study also support the genuine evoked nature of the current changes in coherence. Finally, coherence changes in the gamma-functional connection showed a significant correlation with reaction time, supporting the behavioural relevance of this result and the central role of this functional connection in visual object discrimination in dual task contexts. Future studies performing SPC analysis on single-trial data obtained in similar paradigms may complement current findings and help establishing a more detailed characterization of the brain dynamics supporting dual task performance. While the initial F-statistic threshold for SPC was chosen based on qualitative criteria, the statistical significance of connections of interest is tested by permutation analysis. Thus, the initial thresholding does not undermine the validity of the results in a higher statistical sense. Note that simply lowering the initial F-statistic threshold does not necessarily improve statistical power because if the threshold is chosen too low, spurious pairwise clusters can arise in the permuted data as a matter of chance and thereby reduce power. Future works may also profit from a greater spatial resolution than that provided in the current analysis.

To the best of our knowledge, this study provides the first characterization of the interplay of spatially distinct and functionally specialized brain regions during the execution of a basic perceptual task as a function of concurrent cognitive load. The results highlight the complex functional relationship between frontal-executive and occipitoparietal-perceptual processes and support the central role of dorsal and ventral frontal regions in the modulation of perceptual processes in managing the demands of multiple concurrent goals. In line with recent works [11], our study also demonstrates the value of our method in characterizing large-scale brain functional connections in a data-driven manner using EEG.

## Supporting Information

Figure S1 **Analysis of power as a function of the three visual-spatial working memory (VSWM) load conditions.** Box Plots depicts 50^th^, 75^th^ and 25^th^ percentiles of coherence in each condition. Top and bottom lines extending from the box represents the highest and lowest values of coherence that are not outliers (>1.5 the interquartile range). + represent outlier data.
(TIF)

Figure S2 **Boxplots of coherence values in specific functional connections at different latencies ofinterest.** Box Plots depicts 50^th^, 75^th^ and 25^th^ percentiles of coherence in each condition. Top and bottom lines extending from the box represents the highest and lowest values of coherence that are not outliers (>1.5 the interquartile range). + represent outliers data.
(TIF)

Table S1 **Coordinates of the centroid of cluster activity.** Note: Coordinates of the centroid of cluster activity (x, y, z) are given in Montreal Neurological Institute (MNI) atlas space. Probability value (p-values) are all familywise error (FWE) corrected.
(DOC)

## References

[1] Varela F, Lachaux JP, Rodriguez E, Martinerie J (2001). The brainweb: phase synchronization and large-scale integration.. Nat Rev Neurosci.

[2] Friston KJ (2011). Functional and Effective Connectivity: A Review.. Brain Connectivity.

[3] Pashler H (1994). Dual-task interference in simple tasks: data and theory.. Psychol Bull.

[4] Kim SY, Kim MS, Chun MM (2005). Concurrent working memory load can reduce distraction.. Proc Natl Acad Sci U S A.

[5] Cocchi L, Toepel U, De Lucia M, Martuzzi R, Wood SJ (2011). Working memory load improves early stages of independent visual processing.. Neuropsychologia.

[6] Rissman J, Gazzaley A, D'Esposito M (2009). The effect of non-visual working memory load on top-down modulation of visual processing.. Neuropsychologia.

[7] Hesselmann G, Flandin G, Dehaene S (2011). Probing the cortical network underlying the psychological refractory period: A combined EEG-fMRI study.. Neuroimage.

[8] Sigman M, Dehaene S (2008). Brain mechanisms of serial and parallel processing during dual-task performance.. J Neurosci.

[9] Dux PE, Ivanoff J, Asplund CL, Marois R (2006). Isolation of a central bottleneck of information processing with time-resolved FMRI.. Neuron.

[10] Zalesky A, Fornito A, Bullmore ET (2010). Network-based statistic: identifying differences in brain networks.. Neuroimage.

[11] Hipp JF, Engel AK, Siegel M (2011). Oscillatory synchronization in large-scale cortical networks predicts perception.. Neuron.

[12] Kurylo DD, Pasternak R, Silipo G, Javitt DC, Butler PD (2007). Perceptual organization by proximity and similarity in schizophrenia.. Schizophr Res.

[13] Kubovy M, van den Berg M (2008). The whole is equal to the sum of its parts: a probabilistic model of grouping by proximity and similarity in regular patterns.. Psychol Rev.

[14] Perrin F, Pernier J, Bertrand O, Giard MH, Echallier JF (1987). Mapping of scalp potentials by surface spline interpolation.. Electroencephalogr Clin Neurophysiol.

[15] Grave de Peralta Menendez R, Gonzalez Andino S, Lantz G, Michel CM, Landis T (2001). Noninvasive localization of electromagnetic epileptic activity. I. Method descriptions and simulations.. Brain Topogr.

[16] Spinelli L, Andino SG, Lantz G, Seeck M, Michel CM (2000). Electromagnetic inverse solutions in anatomically constrained spherical head models.. Brain Topogr.

[17] Zalesky A, Fornito A, Harding IH, Cocchi L, Yucel M (2010). Whole-brain anatomical networks: does the choice of nodes matter?. Neuroimage.

[18] Gonzalez Andino SL, Michel CM, Thut G, Landis T, Grave de Peralta R (2005). Prediction of response speed by anticipatory high-frequency (gamma band) oscillations in the human brain.. Hum Brain Mapp.

[19] Gonzalez Andino SL, Murray MM, Foxe JJ, de Peralta Menendez RG (2005). How single-trial electrical neuroimaging contributes to multisensory research.. Exp Brain Res.

[20] Michel CM, Murray MM, Lantz G, Gonzalez S, Spinelli L (2004). EEG source imaging.. Clin Neurophysiol.

[21] Martuzzi R, Murray MM, Meuli RA, Thiran JP, Maeder PP (2009). Methods for determining frequency- and region-dependent relationships between estimated LFPs and BOLD responses in humans.. J Neurophysiol.

[22] Grinsted A, Moore JC, Jevrejeva S (2004). Application of the cross wavelet transform and wavelet coherence to geophysical time series.. Nonlinear Processes in Geophysics.

[23] Smith SM, Miller KL, Salimi-Khorshidi G, Webster M, Beckmann CF (2011). Network modelling methods for FMRI.. Neuroimage.

[24] Palva JM, Monto S, Kulashekhar S, Palva S (2010). Neuronal synchrony reveals working memory networks and predicts individual memory capacity.. Proc Natl Acad Sci U S A.

[25] Schoffelen JM, Gross J (2009). Source connectivity analysis with MEG and EEG.. Hum Brain Mapp.

[26] Schoffelen JM, Gross J (2011). Improving the interpretability of all-to-all pairwise source connectivity analysis in MEG with nonhomogeneous smoothing.. Hum Brain Mapp.

[27] David O, Kiebel SJ, Harrison LM, Mattout J, Kilner JM (2006). Dynamic causal modeling of evoked responses in EEG and MEG.. Neuroimage.

[28] Maris E, Oostenveld R (2007). Nonparametric statistical testing of EEG- and MEG-data.. J Neurosci Methods.

[29] Nichols TE, Holmes AP (2002). Nonparametric permutation tests for functional neuroimaging: a primer with examples.. Hum Brain Mapp.

[30] Lachaux JP, Rodriguez E, Martinerie J, Varela FJ (1999). Measuring phase synchrony in brain signals.. Hum Brain Mapp.

[31] Nolte G, Bai O, Wheaton L, Mari Z, Vorbach S (2004). Identifying true brain interaction from EEG data using the imaginary part of coherency.. Clin Neurophysiol.

[32] Macmillan NA, Creelman CD (2005). Detection Theory: A User's Guide. Second Edition ed..

[33] Badre D (2008). Cognitive control, hierarchy, and the rostro-caudal organization of the frontal lobes.. Trends Cogn Sci.

[34] Dosenbach NU, Fair DA, Cohen AL, Schlaggar BL, Petersen SE (2008). A dual-networks architecture of top-down control.. Trends Cogn Sci.

[35] Koechlin E, Summerfield C (2007). An information theoretical approach to prefrontal executive function.. Trends Cogn Sci.

[36] Corbetta M, Kincade JM, Shulman GL (2002). Neural systems for visual orienting and their relationships to spatial working memory.. J Cogn Neurosci.

[37] Shulman GL, Pope DL, Astafiev SV, McAvoy MP, Snyder AZ (2010). Right hemisphere dominance during spatial selective attention and target detection occurs outside the dorsal frontoparietal network.. J Neurosci.

[38] D'Esposito M, Detre JA, Alsop DC, Shin RK, Atlas S (1995). The neural basis of the central executive system of working memory.. Nature.

[39] Tallon-Baudry C (2009). The roles of gamma-band oscillatory synchrony in human visual cognition.. Front Biosci.

[40] Gazzaley A, Rissman J, Desposito M (2004). Functional connectivity during working memory maintenance.. Cogn Affect Behav Neurosci.

[41] Harrison SA, Tong F (2009). Decoding reveals the contents of visual working memory in early visual areas. Nature.. Nature.

[42] Dreher JC, Koechlin E, Tierney M, Grafman J (2008). Damage to the fronto-polar cortex is associated with impaired multitasking.. PLoS ONE.

[43] Rubens MT, Zanto TP (2011). Characterizing the involvement of rostrolateral prefrontal cortex in prospective memory.. J Neurosci.

[44] Badre D, D'Esposito M (2009). Is the rostro-caudal axis of the frontal lobe hierarchical?. Nat Rev Neurosci.

[45] Koechlin E, Basso G, Pietrini P, Panzer S, Grafman J (1999). The role of the anterior prefrontal cortex in human cognition.. Nature.

[46] Buckley MJ, Sigala N (2010). Is top-down control from prefrontal cortex necessary for visual categorization?. Neuron.

[47] Miller BT, Deouell LY, Dam C, Knight RT, D'Esposito M (2008). Spatio-temporal dynamics of neural mechanisms underlying component operations in working memory.. Brain Res.

[48] Hampshire A, Chamberlain SR, Monti MM, Duncan J, Owen AM (2010). The role of the right inferior frontal gyrus: inhibition and attentional control.. Neuroimage.

