# Supplementary figures and images for: Dynamic Changes in Brain Functional Connectivity during Concurrent Dual-Task Performance
Source: PLoS One. 2011 Nov 29;6(11):e28301. doi: 10.1371/journal.pone.0028301 (PMC3226683; doi:10.1371/journal.pone.0028301)

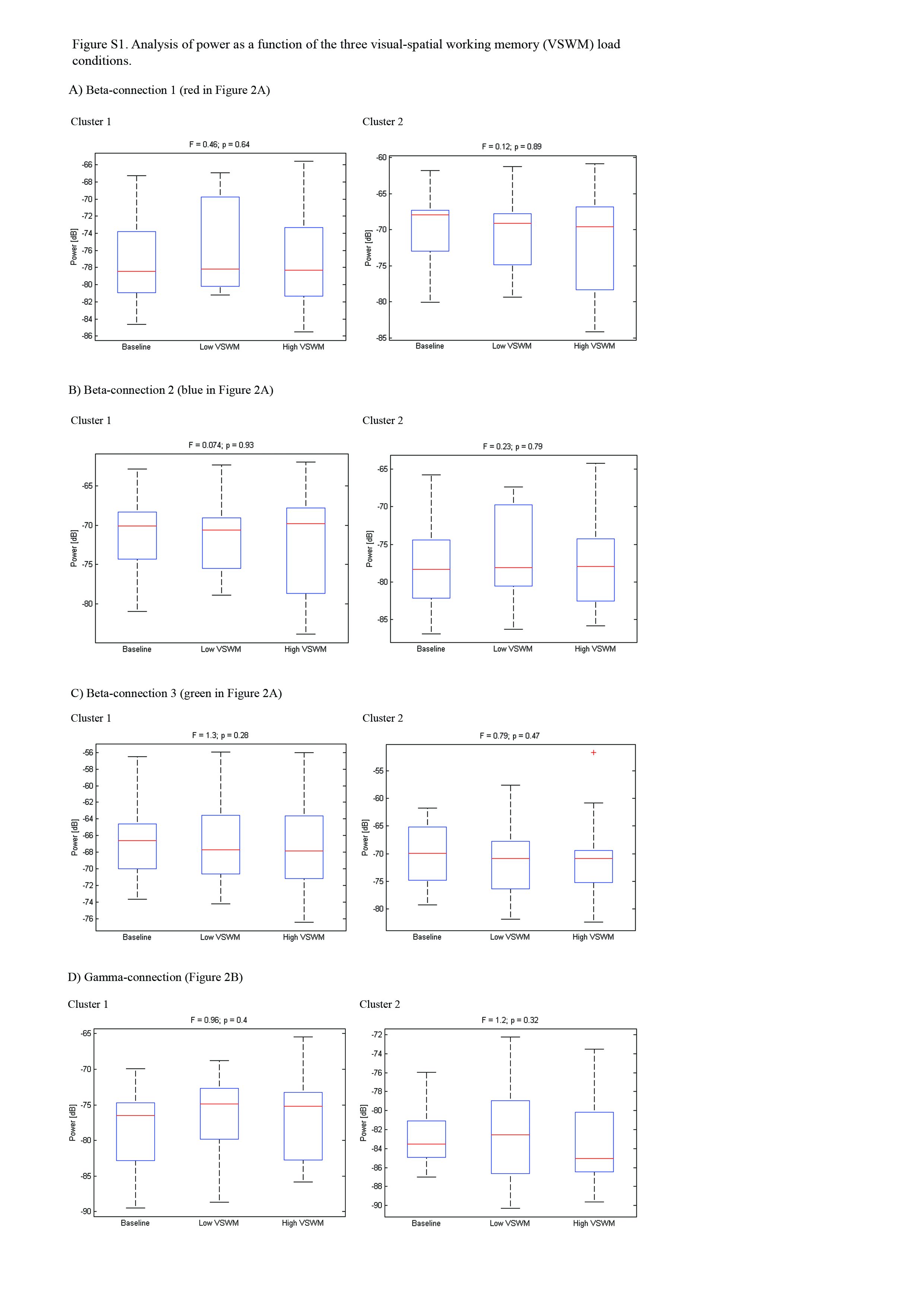

Supplement: Figure S1 — Analysis of power as a function of the three visual-spatial working memory (VSWM) load conditions. Box Plots depicts 50th, 75th and 25th percentiles of coherence in each condition. Top and bottom lines extending from the box represents the highest and lowest values of coherence that are not outliers (>1.5 the interquartile range). + represent outlier data. (TIF) [file pone.0028301.s001.tif]

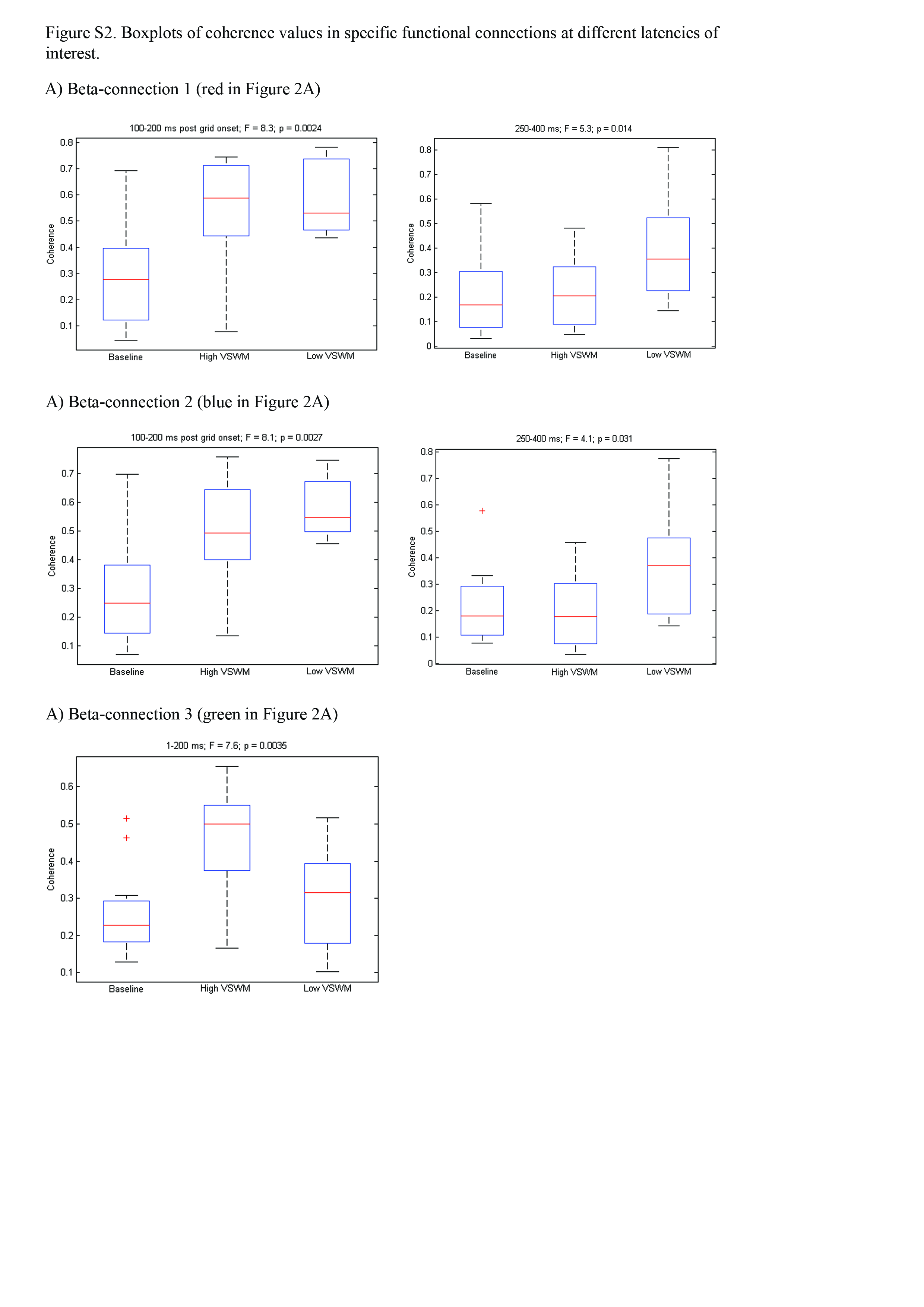

Supplement: Figure S2 — Boxplots of coherence values in specific functional connections at different latencies ofinterest. Box Plots depicts 50th, 75th and 25th percentiles of coherence in each condition. Top and bottom lines extending from the box represents the highest and lowest values of coherence that are not outliers (>1.5 the interquartile range). + represent outliers data. (TIF) [file pone.0028301.s002.tif]
