# Supplementary material for: Dynamic Changes in Brain Functional Connectivity during Concurrent Dual-Task Performance
Source: PLoS One. 2011 Nov 29;6(11):e28301. doi: 10.1371/journal.pone.0028301 (PMC3226683; doi:10.1371/journal.pone.0028301)
Supplement: Table S1 — Coordinates of the centroid of cluster activity. Note: Coordinates of the centroid of cluster activity (x, y, z) are given in Montreal Neurological Institute (MNI) atlas space. Probability value (p-values) are all familywise error (FWE) corrected. (DOC) [file pone.0028301.s003.doc]

**Table S1**

|  | **Anatomical location of the clusters centroid** | **x** | **y** | **z** | **Connections *p-values*** |
| --- | --- | --- | --- | --- | --- |
| **Beta-connection 1** | Gyrus descendens | 36 | -82 | -18 | 0.0115 |
|  | Superior lingual gyrus | 26 | -74 | -16 |  |
|  | Superior frontal gyrus | 18 | 8 | 58 |  |
|  |  | 12 | 18 | 62 |  |
| **Beta-connection 2** | Gyrus descendens | 24 | -88 | -18 | 0.0007 |
|  | Inferior occipital gyrus | 36 | -82 | -18 |  |
|  | Middle occipital gyrus | 44 | -82 | -10 |  |
|  | Middle frontal gyrus | 30 | 14 | 56 |  |
|  |  | 26 | -2 | 60 |  |
|  | Superior frontal gyrus | 18 | 8 | 58 |  |
| **Beta-connection 3** | Cuneus | -14 | -98 | -12 | 0.0408 |
|  |  | -6 | -88 | -4 |  |
|  | Gyrus descendens | -26 | -98 | -10 |  |
|  | Frontopolar gyrus | 24 | 62 | 10 |  |
| **Gamma-connection** | Middle occipital gyrus | 44 | -80 | 4 | 0.0098 |
|  |  | 34 | -92 | 8 |  |
|  |  | 36 | -82 | 16 |  |
|  |  | 44 | -76 | 24 |  |
|  | Posterior orbital gyrus | 46 | 30 | -4 |  |

Note: Coordinates of the centroid of cluster activity (x, y, z) are given in Montreal Neurological Institute (MNI) atlas space. Probability value *(p-values)* are all familywise error (FWE) corrected.
